# Obesity and Body Mass Components Influence Exercise Tolerance and the Course of Hypertension in Perimenopausal Women

**DOI:** 10.3390/jcdd9080238

**Published:** 2022-07-27

**Authors:** Agata Bielecka-Dabrowa, Katarzyna Gryglewska, Agata Sakowicz, Marek Rybak, Kamil Janikowski, Maciej Banach

**Affiliations:** 1 Heart Failure Unit, Department of Cardiology and Congenital Diseases of Adults, Polish Mother’s Memorial Hospital Research Institute (PMMHRI), 93-338 Lodz, Poland; mrybak_1984@tlen.pl (M.R.); kkjanikowski@gmail.com (K.J.); maciejbanach77@gmail.com (M.B.); 2 Department of Preventive Cardiology and Lipidology, Medical University of Lodz, 90-419 Lodz, Poland; 3 Department of Medical Biotechnology, Medical University of Lodz, 90-419 Lodz, Poland; agata.sakowicz@gmail.com

**Keywords:** hypertension, body mass index, troponin, obesity

## Abstract

The aim of this study was to identify the potential influence of obesity and body mass components on exercise tolerance assessed in cardiopulmonary exercise testing (CPET), biochemical and echocardiographic parameters and factors correlated with oxygen absorption at the anaerobic threshold in hypertensive women with low levels of physical activity in the perimenopausal period. The study comprised 188 hypertensive women divided, based on body mass index (BMI), into an obesity group and a non-obesity group. Women with BMI ≥ 30 kg/m^2^ had significantly higher parameters of left ventricular diastolic dysfunction in echocardiography, lower total body water (TBC) in percentage assessed by bioimpedance and significantly worse exercise capacity assessed by CPET. In the study group, VO_2_ AT (mL/kg/min) correlated positively with TBW (r = 0.4, *p* < 0.0001) and with the ratio of extracellular water to total body water (ECW/TBW) (r = 0.4, *p* < 0.00001) and negatively with fat (% and kg) (r = −0.4, *p* < 0.0001 for both). Obesity negatively affects parameters of diastolic left ventricular function, as well as exercise tolerance in CPET in hypertensive females during the perimenopausal period. The oxygen uptake at anaerobic threshold correlates positively with total body water and ECW/TBW and negatively with body fat; this connection is more pronounced in women without obesity. ClinicalTrials.gov Identifier: NCT04802369.

## 1. Introduction

The preponderance of obesity is predicted to reach 21% in women and 18% in men by 2025 [1]. Age is a relevant factor for obesity development. The burden of this condition is highest in those aged 40–64 years [2]. Obesity is one of the principal causes of hypertension, diabetes, dyslipidemia, heart failure, coronary heart disease (CHD) and stroke. Epidemiological and longitudinal studies have documented a strong relationship between obesity and hypertension. Obesity and hypertension are both affiliated with elevated cardiovascular mortality, and they often occur together [3]. In women, hypertension is usually noted after menopause. The main factor is decreased estradiol concentration, which causes deteriorated endothelial function [4]. Hypertension susceptibility in women increases during perimenopause, the transition period before menopause characterized by extended hormone cycles and unstable estrogen fluctuation. Additionally, physiological, social, psychological and affective changes are observed. During this period, female body prepares for menopause from fertile life. [5]. Multiple mechanisms activate the renin–angiotensin–aldosterone system (RAAS) in obesity, including compression of the kidneys and increased sympathetic nervous system (SNS) activation [6]. The adipose tissue excretes adipokines, such as adiponectin, leptin, resistin and interleukin 6. Some studies also suggest the role and the relationship of adipokines in hypertension. In patients with hypertension, higher levels of leptin and lower levels of adiponectin are observed [7,8,9]. Obesity is associated with *numerous* consequences. Excess visceral fat could lead to increased secretion of proinflammatory adipokines, which may promote insulin resistance (IR). IR stimulates muscle catabolism and mitochondrial dysfunction [10]. Furthermore, it has been suggested that muscle fat infiltration causes IR in obese individuals [11]. Additionally, obese people are prone to the loss of muscle mass and strength due to a tendency to be less physically active [12]. The relationship between obesity and left ventricular dysfunction was first noted in the mid-20th century [13]. Clinical and necropsy studies on morbid obesity confirmed the entity of obese cardiomyopathy, regularly leading to congestive heart failure. An overabundance of fat can lead to growth in preload and afterload as a result of hyperdynamic circulation and volume overload [14]. Additionally, obesity may stimulate the effect of blood pressure on left ventricular (LV) mass growth [15]. Therefore, LV dilation and increased LV mass are common in patients with overweight, with both eccentric and concentric LV geometric patterns. In healthy people, peak oxygen uptake (pVO_2_) declines at a rate of 3 to 8% per decade after the age of 30 years, but adaptation for muscle mass considerably mitigates this decline [16]. An inactive lifestyle in the middle-aged females was affiliated with serious menopausal symptoms and obesity [17]. Patients with metabolic syndrome and overweight/obesity who reported adhering to physical activity recommendations achieved higher maximal oxygen uptake (VO_2 max_) in cardiopulmonary exercise testing (CPET), which confirmed the connection of physical performance with the level of activity [18].

The aim of the present study was to identify the potential influence of obesity and body mass components on exercise tolerance assessed in CPET, biochemical and echocardiographic parameters and factors correlated with oxygen absorption at the anaerobic threshold in hypertensive woman during the perimenopausal period and with low levels of physical activity.

## 2. Materials and Methods

### 2.1. Basic Characteristics

A total of 188 female patients in the perimenopausal period with primary hypertension and low levels of physical activity were enrolled in this study. Hypertension was treated in accordance with current ESC/ESH guidelines. There were no significant differences between compared groups with respect to treatment. In our group, a sedentary lifestyle was defined as fewer than three weekly 30 min periods of physical activity. All participants were adults hospitalized in the Department of Preventive Cardiology and Lipidology and the Department of Cardiology and Congenital Heart Diseases between 2018 and 2021. This group was divided into a group of women with an average age of 53 (±8) with a body mass index (BMI) ≥ 30 kg/m^2^ (median, 33.3; IQR: 31.8–35.1) and a group of women with an average age of 52 (±8) with a BMI < 30 kg/m^2^ (median, 25; IQR: 22.9–27.6). The most common method to measure obesity is body mass index, which based on a person’s weight in kilograms and height in meters. A BMI of 18.5 to 24.9 kg/m^2^ is considered a normal weight. Overweight is defined as a BMI of 25 to 29.9 kg/m^2^, and a BMI of 30 kg/m^2^ or greater is considered obese (class 1: 30–34.9 kg/m^2^; class 2: 35–39.9 kg/m^2^; class 3: ≥40 kg/m^2^) [19]. All subjects gave written informed consent to participate in the study. After the informed consent was signed, clinical data of patients participating in the study were collected. The patients underwent a physical examination based on the standard internal medicine protocol. We paid particular attention to inclusion and exclusion criteria during enrolment in the trial. The study was conducted in accordance with the Declaration of Helsinki and was approved by the Polish Mother’s Memorial Hospital Research Institute Bioethics Commission (PMMHRI-BCO.71/2020).

Exclusion criteria:

(a) Diagnosis of heart failure—left ventricular ejection fraction (LVEF) ≤ 50% and signs and symptoms of heart failure or LVEF ≥ 50% with signs and symptoms and raised natriuretic peptides;
(b) Uncontrollable arterial hypertension—systolic blood pressure ≥150 mmHg and/or diastolic blood pressure ≥100 mmHg;
(c) Diagnosis of cardiomyopathy in medical history;
(d) Intracerebral hemorrhage, stroke, transient ischemic attack in medical history;
(e) Past myocardial infarction;
(f) Active systemic infection;
(g) Pregnancy and lactation;
(h) Registered hyperandrogenism, hyperestrogenism, insulin resistance, premature ovarian failure or polycystic ovary syndrome;
(i) Critical hypo- or hyperthyroidism;
(j) Lysosomal storage diseases;
(k) Active autoimmune disorder;
(l) Documented neoplastic process;
(m) Chronic kidney disease (stage IV and V according to the National Kidney Foundation);
(n) Treatment with antiretroviral and cytostatic drugs, glucocorticosteroids or immunosuppressants;
(o) Registered treatment with blood products within the last 6 months, bone marrow transplant or other organ transplant;
(p) Human immunodeficiency virus (HIV), hepatitis B virus (HBV) or hepatitis C virus (HCV) carrier or positive for hepatitis B surface antigen (HBsAg) or antibodies to HCV;
(q) Alcohol and drug abuse;
(r) Surgery or severe trauma within the last month;
(s) Inability of the patient to collaborate and/or provide informed consent to participate in the study;
(t) Patients who did not express their informed consent to participate in the research.

### 2.2. Echocardiography

Quantitative measures were performed in compliance with current guidelines [20]. Comprehensive echocardiography was evaluated using commercially available ultrasound systems (Vivid E95—GE Healthcare, Chicago, IL, USA). The two-dimensional biplane-modified Simpson method from a 4- and 2-chamber view was used to obtain left ventricular volume (LV) and ejection fraction (EF). This method was also necessary to measure maximal left atrial volume (LAV) based on the apical 2- and 4-chamber views at end systole without foreshortening, excluding the LA appendage and pulmonary vein confluences [21]. Each LAV was indexed by body surface area (LAVi). Transmitral velocities, the peak early (E) and peak late (A) mitral velocities, the ratio between the peak E and A velocities (E/A), deceleration time and isovolumic relaxation time were assessed using pulse Doppler echocardiography. Doppler tissue imaging was used to measure early diastolic (E′) velocity and late diastolic (A′) velocity. The ratio of early transmitral peak velocity to early diastolic peak annular velocity (E/E′) was calculated as an index of LV filling pressure. Tricuspid annular plane systolic excursion (TAPSE) measurement was obtained from the conventional measure from the apical four-chamber view [22].

### 2.3. Laboratory Tests

Blood samples were collected into polyethylene–terephthalate tubes containing EDTA. The collected blood samples were mixed well, stored at 4 °C and centrifuged within 6 h. The resulting plasma samples were immediately frozen and stored at −80 °C until measurements were conducted. Laboratory tests were performed in the hospital laboratory following a minimum 12 h period after the last meal. Routine laboratory tests included liver function parameters (aspartate transaminase (ASP) and alanine aminotransferase (ALT)), renal function parameters (creatinine, glomerular filtration rate (GFR) estimated by modification of diet in renal disease (MDRD)) inflammatory cytokine (C-reactive protein (CRP)), glucose level, lipoprotein profile: total cholesterol (TC), low-density lipoprotein (LDL), high-density lipoprotein (HDL) triglycerides (TG), hematology and hypersensitive cardiac troponin T (hsTnT). Serum N-terminal pro-B-type natriuretic peptide (NT-pro-BNP) concentrations were determined using the electrochemiluminescence method.

### 2.4. Spiroergometry

CPET ensures an overall evaluation of the exercise response involving the cardiovascular, pulmonary and skeletal muscle systems. Parameters were measured using the MetaSoft^®^ Studio application (software of CORTEX systems, Leipzig, Germany). Prior to exercise, basic spirometry was performed. We recorded forced vital capacity (FVC) and forced expiratory volume in one second (FEV1). Additionally, the FEV1/FVC ratio (Tiffeneau index) was obtained [23]. Exercise testing in CPET was performed using a stationary cycle ergometer. Heart rate, rhythm, ECG, blood pressure and oxygen saturation were monitored during exercise. The ensuant parameters are relevant to analysis of CPET. The difference between the volume of O_2_ in the inhaled and exhaled air during exercise per unit of time is defined as oxygen uptake (VO_2_). In steady state, VO_2_ is equivalent to metabolic O_2_ consumption. VO_2_ peak represents the highest attainable VO_2_ for a subject. The difference between the volume of CO_2_ in the inhaled and exhaled air during exercise per unit of time is called carbon dioxide output (VCO_2_). The respiratory exchange ratio (RER) corresponds to the gas exchange ratio. We also measured anaerobic threshold (AT) and the minute ventilation/carbon dioxide production slope (VE/VCO_2_ slope) [24].

### 2.5. Sphygmocor

A SphygmoCor 9.0 tonometer (AtCor Medical, Sydney, Australia) enables non-invasive evaluation of central aortic pressure waveforms [25]. This diagnostic method provides measurement of aortic systolic pressure (SP aortic), aortic diastolic pressure (DP aortic) and aortic pulse pressure (PP aortic) [26]. The following parameters of arterial stiffness were also obtained. Augmentation pressure (AP) is the difference in pressure between the two peaks during heart ejection. Pulse wave velocity (PWV) is carotid femoral pulse wave velocity in meters/second (m/s), measured as the distance divided by the pulse transit time. An indication of arterial wave reflection due to arterial system elasticity is defined as the augmentation index (AIx) [27].

### 2.6. Body Mass Analysis

A segmental body composition analyzer (Tanita Pro, Tokyo, Japan) is a device used for non-invasive body mass analysis. This tool measures body mass compartments using the DXA method and bioelectrical impedance analysis (BIA method). A constant current source with a high-frequency current (50 kHz, 90 µA) is used with this device. Electrodes placed on the tips of the toes of both feet supply electric current, which flows into the body parts to be measured. We obtained total and regional fat mass (FM) and fat-free mass (FFM). Total body water (TBW), intracellular water (ICW) and extracellular water were measured. We also examined the relationship between ECW and TBW, defined as the ECW/TBW % ratio [28].

### 2.7. Statistical Analysis

Statistical analysis was carried out using the STATISTICA 13.1 software package (StatSoft, Kraków, Poland). The concordance of normal distribution of all variables was calculated with the Shapiro–Wilk test. To compare 2 groups, the Student’s t-test for continuous variables with normal distribution and the Mann–Whitney U test for non-normally distributed variables were used. The correlations between the analyzed parameters were determined by Spearman’s test. For analyses, a *p*-value < 0.05 was considered statistically significant. The factors that significantly differ between obese and non-obese patients were introduced to the backward step logistic regression model to identify the parameters that best characterize the obese population. The results of logistic regression analysis are presented as odds ratio (OR) and 95% confidence interval (Cl), as well as *p*-value.

## 3. Results

### 3.1. Evaluation of Basic Characteristics

**Figure 1:**
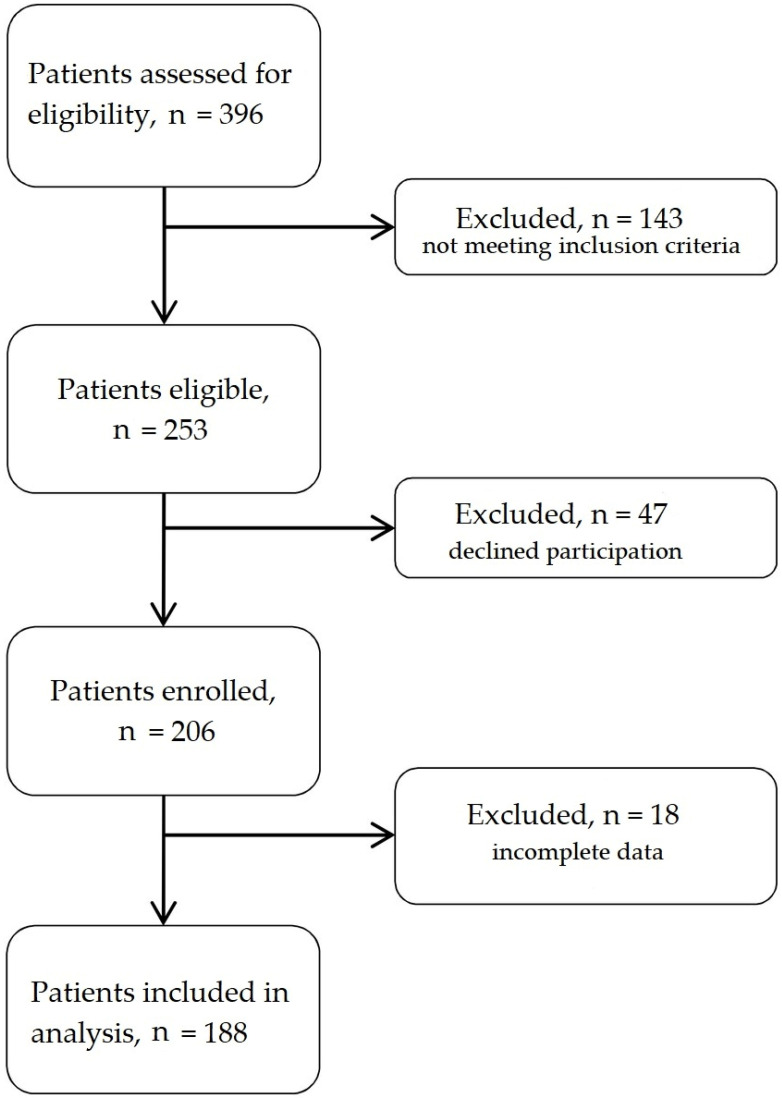
Flow chart of patient enrolment.

A total of 188 female patients were included in the study. Figure 1 shows patient enrolment in the study. The group was divided into two groups: 60 obese women with an average age of 53 (±8) and a body mass index (BMI) ≥ 30 kg/m^2^ (median, 33.3; IQR: 31.8–35.1) and 128 slim women with an average age of 52 (±8) and a BMI < 30 kg/m^2^ (median, 25; IQR: 22.9–27.6). In the obesity group, the blood levels of glucose, triglycerides, ALT, AST, CRP and hsTnT were significantly higher than in the controls group (median, 94 mg/dL; IQR: 89–99 vs. median, 91 mg/dL; IQR: 86–97; *p* = 0.01) (median, 149 mg/dL; IQR: 102–178 vs. median, 111 mg/dL; IQR: 75–146; *p* = 0.002) (median, 23 U/L; IQR: 18–32 vs. median, 17 U/L; IQR: 14.25; *p* < 0.0001) (median, 24 U/L; IQR: 21–30 vs. median, 22 U/L; IQR: 19–25; *p* = 0.02) (median, 0.6 mg/L; IQR: 0.5–0.9 vs. median, 0.5 mg/L; IQR: 0.5–0.5; *p* = 0.004) (median, 4.6 ng/L; IQR: 3–5.9 vs. median, 3.5 ng/L; IQR: 3–4.6; *p* = 0.02), respectively (Table 1). High-density lipoprotein (HDL) cholesterol was lower than in the control group (median, 45 mg/dL; IQR: 38–49 vs. median, 54.5 mg/dL; IQR: 42–66; *p* < 0.0001). There was no important difference in terms of concentration of low-density lipoprotein (LDL) cholesterol, total cholesterol, hemoglobin, glomerular filtration rate (GFR) and N-terminal prohormone of brain natriuretic peptide (NT-proBNP) between women with and without obesity (*p* = 0.3; *p* = 0.9; *p* = 0.2; *p* = 0.2; *p* = 0.2, respectively). Results are shown in Table 1.

### 3.2. Evaluation of Echocardiographic and Hemodynamic Parameters

Left atrial volume (LA volume), left atrial volume index (LAVi), late diastolic filling velocity (A), left atrial volume index over late diastolic mitral annulus velocity (LAVi/A′), and the ratio of peak velocity of early diastolic transmitral flow to peak velocity of early diastolic mitral annular motion (E/E′) were significantly higher (median 66.3 (IQR: 56–82.5) vs. 51 (45.5–62), *p* < 0.0001; median 34 (IQR: 28.8–40.8) vs. 29.6 (IQR: 26.9–34.8), *p* = 0.0005; median 83.5 (IQR: 74.5–93.5) vs. 78 (IQR: 69–90), *p* = 0.03; median 3.4 (IQR: 2.6–4.2) vs. 2.9 (IQR: 2.4–3.4), *p* = 0.005; median 8.5 (IQR: 7.3–10.3) vs. 7.7 (IQR: 6.4–8.6), *p* = 0.004, respectively) in patients with BMI ≥ 30 kg/m^2^ compared to women without obesity. Early diastolic mitral annular velocity (E′) was minor (median 9 (IQR: 8–10.5) vs. 10 (IQR: 8–12), *p* = 0.03) in the study group relative to controls. Groupwise comparisons between ejection fraction (EF), early diastolic filling velocity (E), ratio of early-to-late diastolic transmitral flow velocity (E/A), late diastolic mitral annulus velocity (A′), tricuspid annular plane systolic excursion (TAPSE) and tissue Doppler echocardiography (TDE) were not statistically important. There was no significant difference in pulse wave velocity (PWV), aortic systolic pressure (aortic SP), aortic pulse pressure (aortic PP), augmentation pressure (AP), augmentation index (AIx) or adjusted augmentation index at a heart rate of 75/min (AIx@HR75). Results are presented in Table 2.

### 3.3. Evaluation of Spiroergometry

In basic spirometry, only forced vital capacity (FVC) was statistically lower (median 3.2 (IQR: 2.6–3.5) vs. 3.3 (3–3.6), *p* = 0.04) in the obesity group in comparison to the controls. There was no difference in the remaining parameters: forced expiratory volume in one second (FEV1), the ratio of forced expiratory volume in one second to forced vital capacity (FEV1/FVC), forced expiratory flow over the middle one half of the FVC (FEF 25–75) (*p* = 0.1; *p* = 0.1; *p* = 0.8, respectively). During exercise, maximal peripheral systolic blood pressure (SBP) was greater (median 180 (IQR: 170–200) vs. 160 (160–180), *p* < 0.0001) in patients with BMI ≥ 30 kg/m^2^ than in women without obesity. Differences between maximal peripheral diastolic blood pressure (DBP) were not observed (median 80 (IQR: 80–90), *p* = 0.04). Maximal heart rate was lower (136.3 (±20.4) vs. 136.3 (±20.4), *p* = 0.0006) in obesity patients. Additionally, the respiratory exchange ratio (RER), maximal oxygen consumption measured during incremental exercise indexed per kilogram (VO_2_ max) and oxygen uptake at anaerobic threshold per kilogram (VO_2_ AT) (median 1.1 (IQR: 1–1.1) vs. 1.1 (1.1–1.2); *p* = 0.002; median 18 (IQR: 15–19) vs. 20 (18–23), *p* < 0.0001; median 12 (IQR: 10–13) vs. 13 (11–15), *p* = 0.006m respectively) were lower than in comparison to patients with BMI < 30 kg/m^2^. The highest respiratory oxygen uptake (VO_2_) achieved by the subject during the maximal exercise (peak VO_2_) was greater (median 1.6 (IQR: 1.3–1.7) vs. 1.4 (1.2–1.7), *p* < 0.0001) in the obesity group. However, the minute ventilation/carbon dioxide production slope (VE/VCO_2_ slope) was not much lower (28 (±4.2) vs. 28.6 (±3.9), *p* = 0.4) in patients with BMI ≥ 30 kg/m^2^ compared to controls (Table 3).

### 3.4. Evaluation of Body Mass Analysis

Important differences were observed in all measurements. The level of fat, fat-free body mass (FFM), total body water (TBW), extracellular water (ECW), intracellular water (ICW) and metabolic age were significantly higher (*p* < 0.0001) in patients in the obesity group. Additionally, the ratio of extracellular water to total body water (ECW/TBW%) was greater (*p* < 0.0001) in patients with BMI ≥ 30 kg/m^2^ compared to lower body mass group (Table 4).

### 3.5. Obese Patients and Clinical Parameters: Results of Logistic Regression Analysis

The results of logistic regression analysis indicate a strong relationship between BMI ≥ 30 kg/m^2^ and the following parameters: fat (%), HDL cholesterol (mg/dL), peak VO_2 max_ (L) and VO_2_ max (mL/min/kg) (Table 5).

### 3.6. Significant Correlations with VO_2_ AT

**Figure 2:**
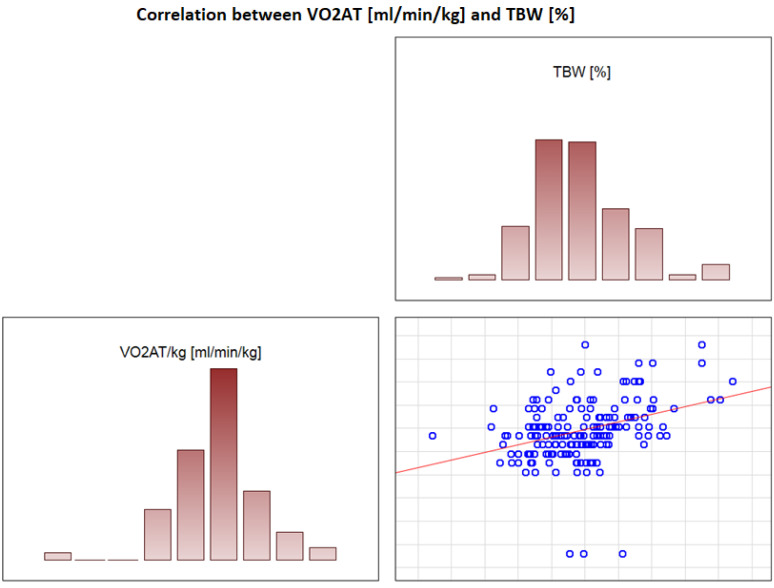
Correlation between VO_2_ AT (mL/min/kg) and TBW (%).

**Figure 3:**
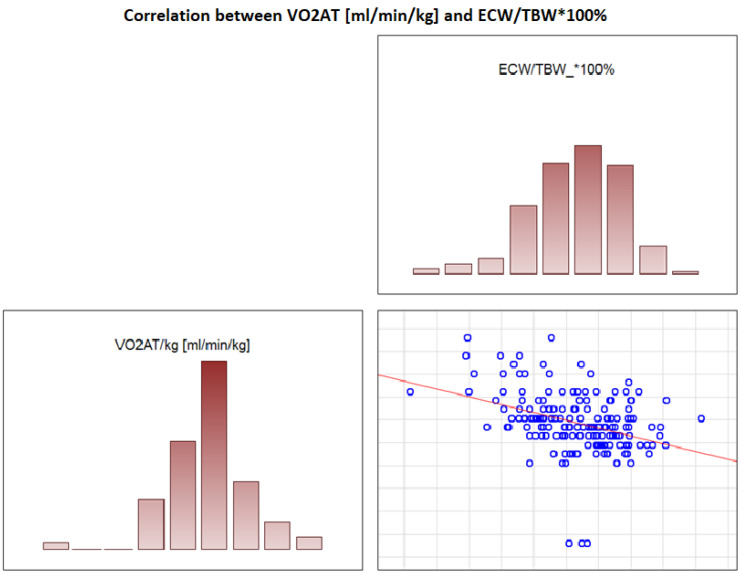
Correlation between VO_2_ AT (mL/min/kg) and ECW/TBW*100%.

**Figure 4:**
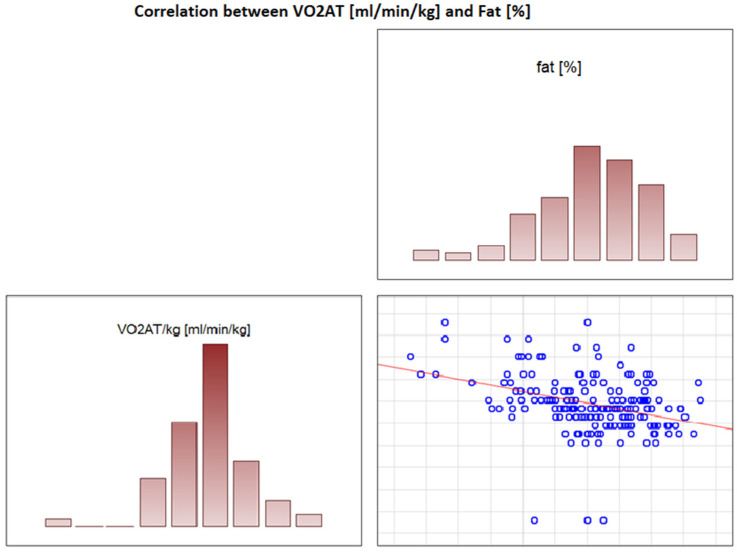
Correlation between VO_2_ AT (mL/min/kg) and fat (%).

**Figure 5:**
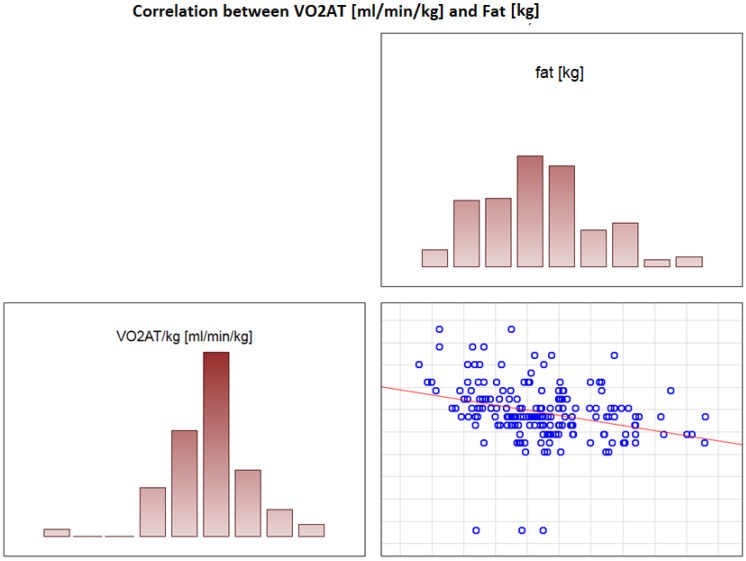
Correlation between VO_2_ AT (mL/min/kg) and fat (kg).

In the overall study population, VO_2_ AT (mL/kg/min) correlated positively with TBW (r = 0.4, *p* < 0.0001) (Figure 2) and ECW/TBW (r = 0.4, *p* < 0.00001) (Figure 3) and negatively with fat both in % and kg (r = −0.4, *p* < 0.0001 for both) (Figure 4 and Figure 5).

In the obesity group, exercise time, peak VO_2_ and VO_2 max_ correlated positively with VO_2_ AT (r = 0.55, *p* < 0.0001; r = 0.57, *p* < 0.0001 and r = 0.69; *p* < 0.0001, respectively). Results are shown in Table 6. In the group with BMI < 30 kg/m^2^, the same parameters (exercise time, peak VO_2_ and VO_2 max_) correlated positively with VO_2_ AT (r = 0.41, *p* < 0.0001; r = 0.43, *p* < 0.0001 and r = 0.72; *p* < 0.0001, respectively). Additionally TBW (%) and VO_2_ AT (r = 0.41, *p* < 0.0001) were positively correlated with VO_2_ AT (r = −0.4, *p* < 0.0001; r = −0.41, *p* < 0.0001), whereas fat (kg), fat (%) and ECW/TBW % were negatively correlated with VO_2_ AT (r = −0.44; *p* < 0.0001) in women without obesity (Table 7).

## 4. Discussion

To the best of our knowledge, the presented study is the first analysis of hypertensive women considering the relations between hydration status and VO_2_ AT. In all study groups and women with BMI < 30 kg/m^2^, VO_2_ AT was correlated positively with total body water (in %) and ECW/TBW ratio and negatively correlated with fat (in % and kg). In obese women, total body water content (in % and kg) was significantly lower, and metabolic age was increased compared to counterparts. The ECW/TBW ratio is managed as an index of the extracellular volume status [29]. The ECW/TBW ratio has been identified in various studies as an indirect measure of overhydration. The relationship between body mass components and respiratory parameters in CPET was investigated in a study by Gruchała-Niedoszytko et al. The authors compared CPET and BIA results in two groups: obese and non-obese subjects. Obese patients achieved lower CPET results for the following factors: VO_2 peak_ (*p* < 0.0001), respiratory exchange ratio (*p* < 0.014), oxygen uptake-to-work rate slope (VO_2_ WR) (*p* < 0.0004), anaerobic threshold heart rate (*p* < 0.0003) and VO_2_ AT (*p* < 0.0002). Significant differences were also observed in fat mass (*p* = 0.01), fat-free mass (*p* = 0.007), resting metabolic rate (RMR) (*p* = 0.007), total body water (*p* = 0.01) and extracellular water (*p* = 0.004) between the groups [30]. In another study, the influence of ECW/TBW and cardiovascular mortality and the connection with cardiac biomarkers in hemodialysis patients was explored [31]. The study included 60 participants, 28 of whom died during the study period. The main cause of death was cardiovascular events (43%). These patients had an increased post-dialysis ECW/TBW ratio (*p* = 0.006) compared to the survivors, whereas for cardiovascular mortality, the only important difference was an increased pre-dialysis ECW/TBW. The ability of post-dialysis ECW/TBW ratio to predict mortality had an area under the ROC curve (AUC) of 0.71 (95% CI, 0.57–0.81; *p* = 0.002).

A study by Zuo et al. indicated that among 95 patients who suffered from end-stage chronic kidney disease on peritoneal dialysis, the strongest correlation was noted between ECW/ICW and reduced exercise capacity (r = −0.63; *p* < 0.001) [32]. In another study, fluid volumes were observed to be higher in obese women, with increased expansion for the extracellular component [33].

Our next finding is that obese females had higher levels of high-sensitivity troponin. Laboratory studies indicate that myocardial injury related to the inflammatory effects of fatty tissue may be one path by which obesity leads to myocardial dysfunction and subsequent heart failure. In a study by Ndumele et al., the authors explored the relationship between obesity and cardiac troponin T measured with a new high-sensitivity assay (hs-cTnT) in 9507 participants. Investigators observed that high BMI is positively correlated with high hs-cTnT with an odds ratio of 2.20 (95% CI: 1.59–3.06) for high hs-cTnT after adjustment [34].

Obesity is independently affiliated with abnormal cardiac remodeling, as well as abnormalities in myocardial contractile function and relaxation. An increased level of epicardial adipose tissue can lead to abnormalities in heart structures (left ventricular enlargement, myocardial fibrosis and atrial inflammation), as well as hemodynamic disturbances [35].

The association between high BMI and decreased LV diastolic function was also described in the research of Russo et al. Their study included 950 subjects. In overweight participants, the risk of diastolic dysfunction was significantly higher (adjusted odds ratio: 1.52, 95% confidence interval: 1.04 to 2.22) in comparison to the control group. Analogous results were observed in obese patients compared to lean subjects (adjusted odds ratio: 1.60, 95% confidence interval: 1.06 to 2.41) [36].

In a study by Pascual et al., the authors explored the effect of isolated obesity on cardiac function in 48 obese and 25 lean women with no other disorders. Subclinical diastolic dysfunction was more frequent among obese participants (*p* = 0.002). Subclinical left ventricular diastolic dysfunction was present in isolated obesity an correlated with BMI [37].

Exercise capacity is a variable parameter that can be influenced by current health status, food and medications but mostly by physical training. Physical effort that uses the full power of aerobic changes (work with VO_2 max_) may take up to 5–8 min. Longer effort requires limitation of intensity in order to extend the work. Efforts that last longer (more than 30 min) are characterized by intensity below the anaerobic threshold level. Therefore, VO_2_ at the anaerobic threshold (VO_2_ AT) best represents actual performance.

Hulens et al. explored differences in exercise capacity between obese and lean women [38]. A total of 225 healthy obese and 81 normal-weight females were included. CPET was performed. In obese participants, VO_2_, achieved load, VE, RER, heart rate and perceived exertion at peak effort were significantly lower in comparison to the non-obese group (*p* = 0.0001). In another study by Gonze et al., the authors analyzed CPETs of 755 obese and 839 non-obese participants. Obese patients achieved the worst results in terms of most of the variables evaluated [39].

The results of multivariable analysis indicate a significant relationship between high body mass index, i.e., ≥30 kg/m^2^, and the following clinical parameters: fat (%) (OR: 1.49; 95% CI: 1.16–1.90; *p* = 0.002), HDL cholesterol (mg/dL) (OR: 0.94; 95% CI: 0.88–0.99; *p* = 0.045), peak VO_2 max_ (L) (OR: 9266.68; 95% CI: 76.22–126,642.67; *p* < 0.001) and VO_2_ max (mL/min/kg) (OR: 0.51; 95% CI: 0.33–0.80; *p* = 0.003), which indicates the negative influence of obesity on exercise tolerance.

The findings of this study should be interpreted in light of some limitations, including the small study population (188 women). The cross-sectional design of this study precludes the opportunity to derive direct cause-and-effect risk associations. We only evaluated patients with mild or moderate arterial hypertension. The results should be carefully elucidated when applied to different populations with HA. The study design was limited with respect to evaluation of the effect of medications. Additionally, the study focused on patients who were capable of performing CPET. Moreover, an echocardiogram was assessed only at rest. These data should be interpreted with caution. Future studies in a larger HA population are therefore recommended.

## 5. Conclusions

Obesity negatively affects parameters of diastolic left ventricular function, as well as exercise tolerance in CPET. Oxygen uptake at anaerobic threshold correlates positively with total body water and ECW/TBW and correlates negatively with body fat; this connection is more pronounced in women without obesity.

## Figures and Tables

**Table 1:** Basic characteristics and biochemical parameters of study groups.

Parameter	BMI ≥ 30 kg/m^2^ *n* = 60	BMI < 30 kg/m^2^ *n* = 128	*p*
Age	53.0 (±8)	52.0(±8)	0.5
Height (cm)	163.5 *, (158–165.5)	164.0 *, (160–167)	0.3
Body mass (kg)	88.3 *, (82.9–97.2)	67.0 *, (61.2–73.2)	<0.0001
BMI (kg/m^2^)	33.3 *, (31.8–35.1)	25.0 *, (22.9–27.6)	<0.0001
Glucose (mg/dL)	94.0 *, (89–99)	91.0 *, (86–97)	0.01
HDL cholesterol (mg/dL)	45.0 *, (38–49)	54.5 *, (42–66)	<0.0001
LDL cholesterol (mg/dL)	118.7 (±32.4)	112.8 (±32.9)	0.3
Triglycerides (mg/dL)	149.0 *, (102–178)	111.0 *, (75–146)	0.002
Total cholesterol (mg/dL)	192.5 (±40.2)	193.0 (±39.5)	0.9
Hemoglobin (g/dL)	13.5 *, (12.2–14.3)	13.4 *, (12.5–14)	0.2
GFR (mL/min/1,73 m^3^)	84.0 *, (74.7–96)	86.9 *, (77.2–100.1)	0.2
ALT (U/L)	23.0 *, (18–32)	17.0 *, (14–25)	<0.0001
AST (U/L)	24.0 *, (21–30)	22.0 *, (19–25)	0.02
CRP (mg/L)	0.6 *, (0.5–0.9)	0.5 *, (0.5–0.5)	0.004
hsTnT (ng/L)	4.6 *, (3–5.9)	3.5 *, (3–4.6)	0.02
NT-proBNP (pg/mL)	65.5 *, (34.5–105)	68.0 *, (44–117.5)	0.2

*—median; BMI—body mass index. Values with non-normal distribution are expressed as median (IQR) values. Values with normal distribution are expressed as mean ± standard deviation (SD). ALT—alanine aminotransferase; AST—aspartate aminotransferase; CRP—C-reactive protein; GFR—glomerular filtration rate; HDL—high-density lipoprotein; LDL—low-density lipoprotein; hsTnT—high-sensitivity troponin; NT-proBNP—N-terminal prohormone of brain natriuretic peptide.

**Table 2:** Evaluation of selected echocardiographic and hemodynamic parameters.

Parameter	BMI ≥ 30 kg/m^2^ *n* = 60	BMI < 30 kg/m^2^ *n* = 128	*p*
LVEF (%)	64.0 *, (61–65.5)	64.0 *, (61–67)	0.8
LA volume (mL)	66.3 *, (56–82.5)	51.0 *, (45.5–62)	<0.0001
LAVi (mL/m^2^)	34.0 *, (28.8–40.8)	29.6 *, (26.9–34.8)	0.0005
E (cm/s)	85.2 (±13.1)	84.5 (±13.6)	0.7
A (cm/s)	83.5 *, (74.5–93.5)	78.0 *, (69–90)	0.03
E/A	1.0 (±0.2)	1.1 (±0.2)	0.1
E′ (cm/s)	9.0 *, (8–10.5)	10.0 *, (8–12)	0.03
A′ (cm/s)	10.0 *, (9.5–12)	10.0 *, (9–12)	0.9
E/E′ (cm/s)	8.5 *, (7.3–10.3)	7.7 *, (6.4–8.6)	0.004
LAVi/A′	3.4 *, (2.6–4.2)	2.9 *, (2.4–3.4)	0.005
TAPSE (mm)	23.0 *, (21–26)	23.0 *, (21–27)	0.8
TDE S′ (cm/s)	16.0 *, (13–17)	14.0 *, (12–16)	0.1
PWV (m/s)	7.4 *, (6.8–8.4)	7.5 *, (6.9–8.5)	0.4
Aortic SP (mmHg)	118.0 *, (112–129)	118.5 *, (109.5–130)	0.9
Aortic PP (mmHg)	67.0 *, (40–79)	66.5 *, (43–81)	0.6
AP (mmHg)	12.0 *, (9–17)	13.0 *, (10–16)	0.7
Alx (%)	31.0 *, (24–37)	33.0 *, (27–38)	0.4
Alx@HR75 (%)	28.0 *, (22–35)	31.0 *, (23–37)	0.3

*—median; BMI—body mass index. Values with non-normal distribution are expressed as median (IQR) values. Values with normal distribution are expressed as mean ± standard deviation (SD). Aortic SP—aortic systolic pressure; aortic PP—aortic pulse pressure; AP—augmentation pressure; AIx—augmentation index; AIx@HR75—adjusted augmentation index at a heart rate of 75/min; A—late diastolic filling velocity; A′—late diastolic mitral annulus velocity; E/A—ratio of early-to-late diastolic transmitral flow velocity; E/E′—ratio of peak velocity of early diastolic transmitral flow to peak velocity of early diastolic mitral annular motion as determined by pulsed wave Doppler; E—early diastolic filling velocity, E′—early diastolic mitral annular velocity; LA—left atrium; LAVi—left atrial volume index; LAVi/A′—left atrial volume index over late diastolic mitral annulus velocity; LV—left ventricle; EF—left ventricular ejection fraction; PWV—pulse wave velocity; TAPSE—tricuspid annular plane systolic excursion; TDE S′—tissue Doppler echocardiography.

**Table 3:** Evaluation of spiroergometric parameters.

Parameter	BMI ≥ 30 kg/m^2^ *n* = 60	BMI < 30 kg/m^2^ *n* = 128	*p*
Exercise time (min)	8.86 (±2.1)	8.42 (±2.2)	0.2
HR max	136.3 (±20.4)	147.1 (±19.1)	0.0006
Peripheral SBP max (mmHg)	180.0 *, (170–200)	170.0 *, (160–180)	<0.0001
Peripheral DBP max (mmHg)	80.0 *, (80–90)	80.0 *, (80–90)	0.04
FEV1 (L)	2.6 *, (2.3–3)	2.7 *, (2.5–3)	0.1
FVC (L)	3.2 *, (2.6–3.5)	3.3 *, (3–3.6)	0.04
FVC%	108.0 *, (97–117)	109.0 *, (99–122)	0.2
FEV1/FVC	85.0 *, (79–88)	84.0 *, (79–87)	0.1
FEV1/FVC%	107.0 *, (99–111)	105.0 *, (99–109)	0.1
FEF 25–75	2.5 *, (1.8–3.4)	2.6 *, (1.9–3.2)	0.8
RER	1.1 *, (1–1.1)	1.1 *, (1.1–1.2)	0.002
VO_2 max_ (mL/min/kg)	18.0 *, (15–19)	20.0 *, (18–23)	<0.0001
VO_2_ AT (mL/min/kg)	12.0 *, (10–13)	13.0 *, (11–15)	0.006
Peak VO_2 max_ (L)	1.6 *, (1.3–1.7)	1.4 *, (1.2–1.5)	<0.0001
VE/VCO_2_ slope	28.0 (±4.2)	28.6 (±3.9)	0.4

*—median; BMI—body mass index. Values with non-normal distribution are expressed as median (IQR) values. Values with normal distribution are expressed as mean ± standard deviation (SD). DBP—diastolic blood pressure; SBP—systolic blood pressure; FEV1—forced expiratory volume in one second; FVC—forced vital capacity; FEV1/FVC—ratio of forced expiratory volume in one second to forced vital capacity; FEF 25–75%—forced expiratory flow over the middle half of the FVC; RER—respiratory exchange ratio; VO_2_ max—maximum amount of oxygen the body can utilize during a specified period of usually intense exercise; VO_2_ AT—oxygen uptake at anaerobic threshold per kilogram; peak VO_2_—highest respiratory oxygen uptake (VO_2_) achieved by the subject during maximal exercise; VE/VCO_2_ slope—minute ventilation/carbon dioxide production slope.

**Table 4:** Evaluation of body composition parameters.

Parameter	BMI ≥ 30 kg/m^2^ *n* = 60	BMI < 30 kg/m^2^ *n* = 128	*p*
Fat (%)	40.5 *, (37.6–42)	33.3 *, (29.2–36.6)	<0.0001
Fat (kg)	36.6 *, (31.3–40.3)	23.0 *, (17.5–26.8)	<0.0001
FFM (kg)	53.8 *, (50.8–56.8)	44.8 *, (42.1–47.2)	<0.0001
TBW (kg)	38.3 *, (36–40.4)	31.9 *, (30–33.6)	<0.0001
TBW (%)	42.2 *, (41.4–44.4)	47.4 *, (44.9–50.4)	<0.0001
ECW (kg)	17.6 *, (16.8–18.7)	14.3 *, (13.6–15.2)	<0.0001
ICW (kg)	20.5 *, (19.2–22.3)	17.5 *, (16.2–18.9)	<0.0001
ECW/TBW %	46.5 *, (45.6–47.3)	45.1 *, (43.9–46.2)	<0.0001
Metabolic age	64.0 *, (58–70)	47.0 *, (40–55)	<0.0001

*—median; BMI—body mass index. Values with non-normal distribution are expressed as median (IQR) values. Values with normal distribution are expressed as mean ± standard deviation (SD). ECW—extracellular water; FFM—fat-free body mass; ICW—intracellular water; ECW/TBW%—ratio of extracellular water to total body water; TBW—total body water.

**Table 5:** Results of logistic regression analysis.

BMI ≥ 30 kg/m^2^			
Parameter	OR	95% Cl	*p*
Fat (%)	1.49	1.16–1.90	0.002
HDL cholesterol (mg/dL)	0.94	0.88–0.99	0.045
Peak VO_2 max_ (L)	9266.68	76.22–126,642.67	0.001
VO_2 max_ (mL/min/kg)	0.51	0.33–0.80	0.003

HDL—high-density lipoprotein; peak VO_2_—highest respiratory oxygen uptake (VO_2_) achieved by the subject during maximal exercise; VO_2 max_—maximum amount of oxygen the body can utilize during a specified period of usually intense exercise.

**Table 6:** Correlations with VO_2_ AT/kg in patients with BMI ≥ 30 kg/m^2^.

BMI ≥ 30 kg/m^2^		
Parameter	r	*p*
Exercise time (min)	0.55	<0.0001
Peak VO_2 max_ (L)	0.57	<0.0001
VO_2 max_ (mL/min/kg)	0.69	<0.0001

**Table 7:** Correlations with VO_2_ AT/kg in patients with BMI < 30 kg/m^2^.

BMI < 30 kg/m^2^		
Parameter	r	*p*
Exercise time (min)	0.41	<0.0001
Peak VO_2 max_ (L)	0.43	<0.0001
VO_2 max_ (mL/min/kg)	0.72	<0.0001
TBW (%)	0.41	<0.0001
Fat (kg)	−0.4	<0.0001
Fat (%)	−0.41	<0.0001
ECW/TBW %	−0.44	<0.0001

## Data Availability

Individual participant data that underlie the results reported in this article after deidentification (text, tables, figures and appendices), as well as study protocol, will be available for researchers who provide a methodologically sound proposal. Proposals may be submitted after 9 months and up to 36 months following article publication.
